# Reconstruction of Heart-related Imaging from Lung Electrical Impedance Tomography Using Semi-Siamese U-Net

**DOI:** 10.2174/0115734056408077250610070821

**Published:** 2025-07-02

**Authors:** Yen-Fen Ko, Yue-Der Lin, Po-lan Su

**Affiliations:** 1 Department of Biomedical Engineering, College of Biomedical Engineering, China Medical University, Taichung, Taiwan; 2 Department of Automatic Control Engineering, Feng Chia University, Taichung, Taiwan; 3 Department of Internal Medicine, National Cheng Kung University Hospital, College of Medicine, National Cheng Kung University, Tainan, Taiwan

**Keywords:** Electrical impedance tomography, Semi-Siamese U-Net, Lung imaging, Heart imaging, ICU settings, PEEP, ARDS

## Abstract

**Introduction:**

Electrical Impedance Tomography (EIT) is widely used for bedside ventilation monitoring but is limited in reconstructing cardiac-related signals due to the dominance of lung impedance changes. This study aims to reconstruct heart-related impedance imaging from lung EIT using a novel semi-Siamese U-Net architecture.

**Methods:**

A deep learning model was developed with a shared encoder and two decoders designed to segment lung and heart regions independently. The model was trained and validated on FEM-based EIT simulations and tested on real human EIT data. A weighted binary cross-entropy loss was applied to emphasize cardiac-related learning.

**Results:**

The model achieved a Dice coefficient >0.99 and MAE <0.1% on simulation data. It successfully separated lung and heart regions on human EIT frames without additional fine-tuning, demonstrating strong generalization capacity.

**Discussion:**

These findings reveal that the semi-Siamese U-Net can overcome signal dominance and improve cardiac-related EIT reconstruction. However, promising results are currently limited to qualitative evaluation of real data and simulation-based training.

**Conclusion:**

The proposed method offers a potential pathway for simultaneous lung-heart monitoring in ICU settings. Future work will focus on clinical validation and real-time implementation.

## 1 INTRODUCTION

Lung Electrical Impedance Tomography (EIT) is a technique that has rapidly developed in the recent decade and is based on applying alternating micro-currents at 20-50 Hz using a set of electrodes positioned around the chest of the subject. Lung EIT is particularly useful in monitoring the functioning of the lungs because lung tissue conductivity is much lower than most tissues of the organs in the thorax. This leads to high absolute contrast of lung EIT imaging [1]. In clinical practice, Electrical Impedance Tomography (EIT) is typically performed non-invasively using a rubber electrode belt equipped with 16 electrodes, which is placed around the patient's chest. The system operates by applying small alternating electrical currents through the electrodes and measuring the resulting voltage differences. These data are then used to reconstruct real-time images of impedance changes within the thorax, primarily reflecting ventilation patterns in the lung tissue. This technique enables continuous, bedside monitoring of regional lung ventilation without the use of ionizing radiation or invasive procedures. During breathing, the electrically insulating inhaled and exhaled air produces conductivity changes, which EIT can also detect. Among the medical imaging technologies, lung EIT fits the requirement to observe and distinguish between breathing in and out and is particularly well-adapted for bedside use. Thus, since 2011, it has been apparent from early research on lung EIT that imaging of ventilation is a key clinical application of EIT, especially for the physiological evaluation of the critically ill. Canet and Gallart [2] commented that “pulmonary imaging monitors that are easy to use have arrived and will soon be commonplace in operating rooms, revealing the dark side of the lung we have been unable to see until now.”

Thereafter, several studies focused on lung ventilation have been published [3]. These include applications in anesthesiology and the respiratory intensive care unit, in which monitoring of the airway is critical in the operating room and where optimal Positive End-Expiratory Pressure (PEEP) strategies are essential for patients with Acute Respiratory Distress Syndrome (ARDS). In the meantime, a succession of proposed EIT-guiding optimized PEEP strategies have been inconclusive until now [4, 5].

In recent years, researchers and clinicians have become aware of the increasingly important role of perfusion. Perfusion information reflects pulmonary vascular resistance to show systemic circulation [6-8]. Ventilation-perfusion mismatch is the main cause of lack of oxygen and hypoxemia in patients with ARDS or other lung diseases.

Recently, EIT has been used as a bedside monitoring tool to observe the distribution of lung impedance changes during ventilation therapy. Many studies used EIT to guide PEEP and to optimize strategies for individual needs [9, 10]. Most clinical studies on the lung focus on ventilation-induced impedance changes; however, few focus on perfusion-induced impedance changes.

In obtaining lung EIT measurements, both the respiratory-related and cardiovascular-related impedance changes can be measured and gathered simultaneously. Thus, electrical impedance images contain both ventilation and pulmonary blood perfusion-related information. However, it is a challenge to separate the weaker perfusion-related impedance changes compared to the ventilation-induced impedance changes [11, 12]. Graf and Riedel pointed out the issue of significant differences in cardiac-related impedance amplitude; however, these changes have not yet been elucidated, and little explanation has been proffered [13] due to the technical limitation of obtaining ground cardiac-related EIT images.

Based on Table **1**, the resistivity values of various chest tissues (*e.g*., blood, heart, lung at different respiratory states, and fat) show significant variability. This variation is critically important in the context of EIT—a non-invasive imaging technique that reconstructs the conductivity distribution within the thorax based on surface electrode voltage measurements.

In EIT, the internal electrical properties (conductivity) of tissues affect how injected current propagates through the body. Since different tissues have distinct resistivities, they contribute differently to the voltage patterns measured on the surface. This enables the reconstruction of an image reflecting the internal structure and function.

Due to the EIT measuring principle, the electrical properties of tissues and the anatomical arrangement of the internal organs in the thorax lead to the insensitivity of EIT image reconstruction in the central region, which is far from the measuring electrodes. In other words, from the signal point of view, the level of perfusion-related impedance changes is significantly smaller in amplitude compared to the changes induced by ventilation (Table **1**).

Therefore, previous studies proposed several extracting or separating methods based on signals or image processing, such as apnea [14, 15], breath-holding, injecting a contrast agent [1, 2], ECG-gating [4, 6, 7, 16], time domain-based processing, and frequency domain-based processing [17-19]. However, these approaches have limitations that make them unsuitable for providing apparent and rapid response outcomes as auxiliary diagnostics for clinical decision-making. Several studies on separating ventilation and perfusion-related information from EIT data streams in EIT applications have been published. These studies have produced incomplete results and which method is more reliable remains inconclusive. It is a crucial step for an EIT technique to become a routine modality in clinical settings to help physicians make decisions during respiratory therapy. Previous methods have been directly applied to real EIT data to separate ventilation and perfusion-related information, but very few studies have reported simulations and phantom experiments to address this issue. In real EIT applications, it is difficult to consider all the possible situations and distinguish the cause of impedance changes, such as noise and movement.

To address this issue, a Finite Element Method (FEM) phantom was designed to simulate the lung EIT activity, thereby modeling the EIT problem. From the perspective of the reconstructed image, the effectiveness and feasibility of the proposed approach were explored, thereby generating distinguishing perfusion and ventilation information from it.

For these reasons, this study designed a specialized architecture to tackle the challenge of EIT imaging that includes a Deep Neural Network (DNN) and U-Net architecture to generate a non-linear model tailored to learn separation. A succession of studies based on FEM simulation in lung EIT have been published, and a model was tailor-built [20]. Furthermore, the improvement in spatial resolution of ventilation-induced EIT images with real EIT data—which validated the effectiveness of the proposed architecture—was published [21]. The aim of this study was to further demonstrate the reconstruction of the heart-induced EIT image from the original lung EIT image using the proposed architecture.

The contributions of this study are summarized as follows:

1. Based on the state-of-the-art U-Net, a specialized model was trained using the proposed novel architecture semi-Siamese U-Net [20].

2. The optimal parameter of multi-task weighted losses was determined for heart imaging.

3. The learned spatial cardiac-related conductivity distribution was validated *via* FEM-based phantom experiments, demonstrating successful signal separation.

The novelty of the proposed semi-Siamese U-Net lies in its dual-decoder architecture, which is specifically designed to disentangle spatially overlapping signals from the lung and heart within a single EIT image. Conventional U-Net models utilize a single decoder, which may force a compromise between two segmentation tasks when the signal amplitudes differ significantly. By contrast, this semi-Siamese architecture shares a common encoder to extract contextual thoracic features but separates learning paths in the decoders—one optimized for high-amplitude lung features, the other for low-amplitude heart features. This structure improves signal isolation, training stability, and segmentation fidelity, particularly for weak cardiac-related impedance signals that are typically suppressed in standard EIT reconstructions.

This paper is organized as follows. The Materials and Methods section describes the proposed semi-Siamese U-Net model and its key structure design for the multi-segmentation tasks in EIT. The implementation of the FEM-based phantoms for EIT simulation is outlined with an explanation of how the training datasets are produced. The experimental procedure to determine the optimal parameters for reconstructing heart-related EIT imaging is also described. The Results section presents public real human EIT data, a sequence of 24 EIT images of a healthy subject provided by EIDORS, and the corresponding heart reconstruction results.

## 2 MATERIALS AND METHODS

### 2.1 Deep-learning Approach for EIT Heart Image Reconstruction

**Fig. (1):**
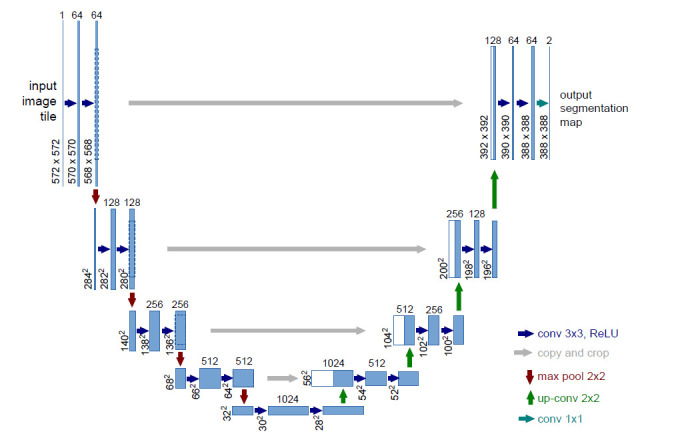
Classical-de factor U-Net architecture [22].

In 2015, Ronneberger *et al*. [22] proposed the U-Net architecture, which is an ingenious convolutional network for biomedical image segmentation. Until now, the classical architecture has become the *de facto* standard biomedical image segmentation model (Fig. **1**). It has advanced in the frontier of semantic segmentation and medical image reconstruction, and has demonstrated breakthrough performance [23-27]. The design of U-Net architecture concatenates the contracting and expanding pathways by the mean of skip connections [28, 29], which uses powerful convolutional neural networks and deep learning to improve medical image quality. The classical architecture was made to effectively capture multi-level semantic information and retain the coarse as well as finer feature information simultaneously [23, 30]. This approach effectively addresses the issue of the EIT ill-posed inverse problem [13] and its application in the study. The lung impedance changes dominate the EIT imaging, and heart-induced impedance changes are too small to be observed. In other words, the EIT imaging of heart-induced impedance changes is a significantly more challenging task than imaging the lung.

**Fig. (2):**
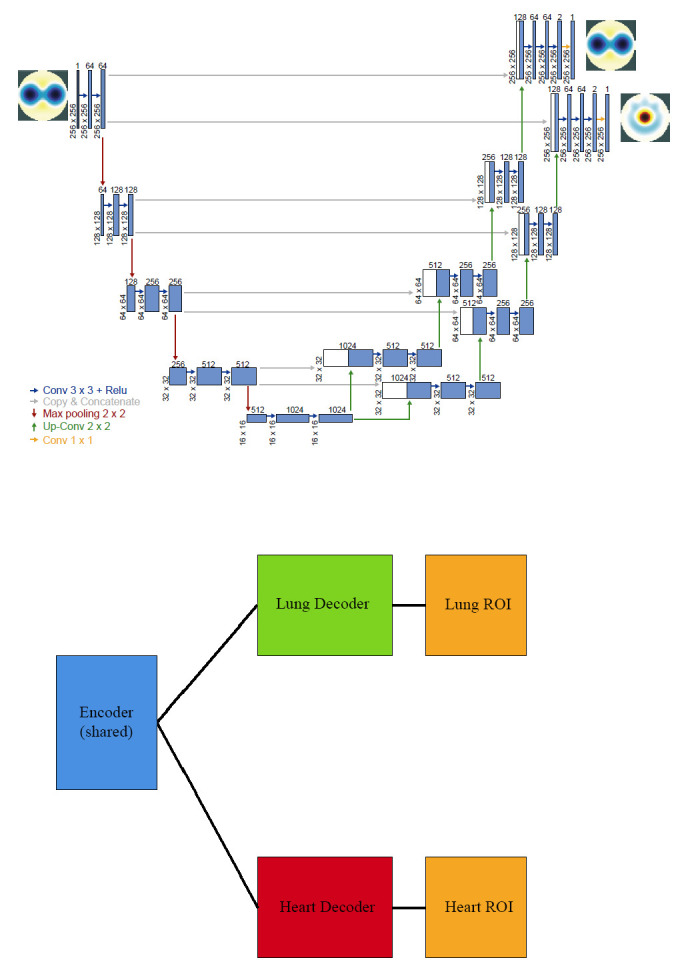
The tailored semi-Siamese U-Net architecture for EIT. The model features a shared encoder (blue) that extracts common thoracic features, followed by two specialized decoders: one for lung segmentation (green) and one for heart segmentation (red). Each decoder performs upsampling, concatenation, and convolution operations to refine features independently. The final outputs are binary masks corresponding to lung and heart regions.

For this reason, a tailored, specialized architecture—semi-Siamese U-Net [20, 21]—was established in this study by mimicking the U-Net concept (Fig. **2**). The specialized architecture comes with multi-weighted losses. Based on this reliable model, the study focused on determining the optimal multi-weighted losses to ensure rigorous training to improve the EIT heart imaging quality under low heart impedance. The specialized architecture with multi-weighted losses design is described in the next section.

The semi-Siamese U-Net model was trained using the FEM-based simulation EIT data as training and validation datasets, and the trained optimal semi-Siamese U-Net model was directly applied to real human data that was not used in training.

### 2.2 Network Architecture

The classical U-Net design used for biomedical image segmentation is constructed of a one-encoder-decoder network, which is concatenated by symmetric layers. Based on the design for semantic information from deep-learning architecture, it learns the context *via* lower layers and precisely localizes the target region *via* higher layers [28].

This study adopted the semi-Siamese U-Net with U-Net design characteristics and advantages by modifying the architecture to a one-encoder, two-decoder network. Accordingly, two decoders form two pathways that yield two individual feature maps for predicting the segmentation masks. This design allows the model to independently learn features for heart and lung tasks, reducing interference caused by their different signal magnitudes and spatial overlaps. The dual-decoder structure is particularly important for cardiac EIT segmentation, which requires amplification and refinement of weak impedance signals that are otherwise dominated by ventilation-related signals. Thus, the semi-Siamese U-Net model enables task-specific learning by using separate decoders for lung and heart image reconstruction. This design allows each decoder to specialize in learning features at appropriate spatial and signal scales, which is essential for isolating the weaker heart-induced impedance changes from the dominant lung signals in EIT data.

Figs. (**1** and **2**) show the schematic overview of the classical U-Net and the tailored semi-Siamese U-Net, respectively. Comparing these two deep networks, the structural modifications of semi-Siamese U-Net provide it advantages over U-Net.

An annotated schematic of the semi-Siamese U-Net architecture is shown in Fig. (**2**), illustrating the shared encoder and dual-decoder branches designed for independent lung and heart segmentation. This structural design supports signal separation by allowing each decoder to specialize in its respective spatial and frequency domains. The dual-path design enhances the model’s ability to isolate low-amplitude cardiac signals while preserving dominant ventilation patterns.

Herein, the structure of the semi-Siamese U-Net is outlined briefly, and the pseudocode is provided below (interested readers may refer to our publications for further details of the mechanism, design, and experiments) [20, 21]. Table **2** summarizes the key design of the semi-Siamese U-Net network, including two main functions.

**# Semi-Siamese U-Net pseudocode**

def semi_siamese_unet(input_image):

features = encoder(input_image) # Shared encoder

lung_output = decoder_lung(features) # Decoder 1

heart_output = decoder_heart(features) # Decoder 2

return lung_output, heart_output

**# Training with multi-weighted loss**

L*lung* = BCE(lung_output, lung_gt)

L*heart* = BCE(heart_output, heart_gt)

Loss_total = W*lung* * L*lung* + W*heart* * L*heart*

### 2.3 Multi-weighted Loss for Heart Imaging Learning

A new loss function (1) was adopted that uses multi-weighted losses, in which the multi-weighted losses, W_lung_ and W_heart_, optimize EIT image reconstruction performance synchronously.


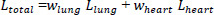
(1)

A pair of datasets of original EIT images and desired reconstructed images were used to train the tailored semi-Siamese U-Net network utilizing stochastic gradient descent of Keras running on top of TensorFlow. The energy function *E* is calculated using a pixel-wise sigmoid activation function over the terminal feature map merged with a binary cross-entropy loss function.

The sigmoid function is defined as:


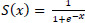
(2)

where *x* denotes the activation in the terminal feature map at each pixel position, *i.e*., *S*(*x*) ≈ 1 has the maximum activation and *S*(*x*) ≈ 0 has the minimum activation.

The binary cross-entropy loss function is defined as:


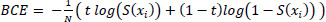
(3)

Where *t* is the ground truth (desired image) and *S*(*x_i_*) is the predicted probability for all N points, which is precomputed using the function (2). Combining (1) with (2) and (3), the binary cross-entropy penalizes the heart-loss function (rigorous heart learning), which is described as:


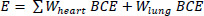
(4)

where *W_heart_* and *W_lung_* are weight constants and *E* denotes the new energy function, *i.e*., *E* ≈ 0 which represents the predicted reconstructed impedance image that approximates the ground truth.

BCE was chosen due to its stability in pixel-wise binary classification tasks, particularly for sparse signals like heart impedance changes. Dice loss was tested but yielded unstable gradients during training.

The convergence of the model minimizes the energy function *E*, which is the corresponding multi-task loss *L_total_* calculated using (1). This optimization enhances the separation performance and compensates for the recessive bioelectric properties of the heart-related EIT image. Based on the two concepts of multi-task and weighted losses shown in Table **2**, new losses were attempted to be parameterized using different ratios of weight constants, which refer to the hyperparameters described in Table **3**.

### 2.4 FEM-based Imaging for EIT Simulations

Sophisticated phantom models were generated using the FEM modeling software Netgen 5.3, and the forward and inverse problems were solved using EIDORS v3.8 to reconstruct and display EIT images. NETGEN is an automatic 3D tetrahedral mesh generator. It accepts input from constructive solid geometry or boundary representation from STL file format. In this way, the phantom built is computable and quantified, apart from the corresponding reconstruction EIT images, and can be used as a training dataset and efficient validating dataset.

#### 2.4.1 FEM Phantom

Fig. (**3**) shows and Table **4** describes the FEM-based phantom for thorax EIT simulation consisting of a 16-electrode ring surrounding the thorax phantom with a pair of drive 1 mA current and the conductivities of spheres referred to in literature [31-33]. All the phantoms were programmable and the radii of spheres were varied from 0.3–0.6 and 0.1–0.3, respectively (unit: arbitrary) for simulation of the thorax activities. The corresponding EIT images and pair dataset were reconstructed and obtained by solving numerical problems: forward and inverse problems using EIDORS.

The deep learning model based on U-Net architecture is a well-known supervised learning method. Therefore, the desired images, including the lung and heart, were required as model learning targets, as shown in Fig. (**4e** and **f**). Fig. (**4**) shows the pair of phantoms Fig. (**4a** and **c**) that were built and the EIT image reconstruction performed to yield pairs of images Fig. (**4d** and **f**) for the training and validation datasets.

#### 2.4.2 Dataset Preprocessing

FEM-based EIT simulation data: Before training, the pair dataset was split into two subsets: (i) training validation and (ii) test sets. The test set was an independent dataset not used in training. Split ratio: 90% training / 10% validation (of 1000 samples). Test set: 200 independent samples. The k-fold cross-validation was used in this experiment.

Real human data: The public EIT data of a healthy adult provided by EIDORS was used to test the ability of the model to reconstruct EIT heart imaging.

The public data are available from the EIDORS website: https://sourceforge.net/p/eidors3d/code/HEAD/tree/trunk/eidors/sample_data/montreal_data_1995.mat.

Semi-Siamese U-Net network training for EIT: The pair datasets were used to train the semi-Siamese model with the following dependencies, hyperparameters, and implementation. The experiments were designed based on multi-weighted loss, and the values of multi-weighted losses were parameterized as the hyperparameters of the model.

Hyperparameters: A new loss function was designed to optimize EIT image reconstruction, especially for reconstruction of the EIT heart imaging. As mentioned before, the novel architecture consists of a duo-encoder structure, *i.e*., a dual pathway to perform the two learning tasks under different scales. The symmetric layers extract spatial information from the original EIT image through concatenation operation.

The new loss function was developed from cross-entropy (4) with weight constants W_heart_ and W_lung_. The weight constants were parameterized into one hyperparameter as a multi-weighted loss function. Experiments were performed using different combinations of weight constants, thereby revealing the interaction in lung and heart imaging reconstructions and tradeoffs between lung and heart reconstructions. The hyperparameters are summarized in Table **3**.

W_lung_ and W_heart_ were initialized to 1 and varied to determine optimal separation tradeoffs. Hyperparameter tuning was carried out *via* grid search over [1.0, 1.5, 1.8, 2.0].

Experimental environment: The experiments were implemented using a server with Intel Core i7-9700K processor and NVIDIA GeForce GTX 1080 Ti GPU. The programming and model were implemented using Keras 2.3.1 in Python 3.6.8.

#### 2.4.3 Evaluation Metrics

Dice similarity coefficient (DSC)— also known as the Sørensen–Dice index or Dice score—and Mean Absolute Error (MAE) were used to evaluate the EIT image reconstruction performance. Dice scores the model learning ability in terms of segmentation precision, and MAE scores the model learning ability for each pixel. These metrics were used because the model learning considers segmentation precision and difference in impedance level, which DSC and MAE measure, respectively. The metrics follow the original semi-Siamese U-Net design [20].

The baseline model, the *de factor* U-Net model architecture, was implemented, and its performance was compared with that of the proposed customized heart-imaging model, semi-Siamese U-Net.

## 3 RESULTS

The semi-Siamese U-Net model was implemented with the above dependencies and hyperparameters. Subsequently, the segmentation results regarding heart-related impedance changes are presented as: FEM-based EIT simulation and real human EIT data application. The FEM-based EIT simulation shows the optimal weight for the heart imaging under multi-segmentation tasks, applying the optimal Wc to implement the heart imaging reconstruction in real human EIT data. The results of lung-induced impedance change and the ventilated lung region for automatic lung regions of interest segmentation in EIT image were published in a study by Ko *et al*. [21].

### 3.1 FEM-based EIT Simulation

Multi-weighted loss: The results of the semi-Siamese U-Net model with the parameterization of new weighted losses and varied heart weight in the loss function are summarized and compared to the baseline model, U-Net, in Table **5**.

Wc represents the weighted constant ratio of W_heart_ to W_lung_ for convenient reading. It is evident that the performance of the heart-related imaging reconstruction with increased Wc is superior. In other words, the semi-Siamese U-Net model enhances the reconstruction of heart imaging by restricting heart imaging errors, as shown in (4). Between Wc from 1.0 to 2.0, the relative improvements show that this semi-Siamese U-Net was superior to the baseline model, which can be attributed to the novel architecture of the tailored semi-Siamese U-Net. Notably, when Wc was increased to 1.5, the performance reached under 1% of MAE, which is 0.69, and further reached under 0.1%, which is 0.056%. Dice score was over 99% at 1.8 of Wc, which continuously achieved 11.53 relative improvement to 99.9%. This can be attributed to the weighting of the heart region in the new loss function.

Performance trade-offs for selection weight constants for hyperparameter: The results of introducing the weighted losses in the semi-Siamese U-Net demonstrated satisfactory performance when measured using DSC and MAE. In addition, the performance progression of the model was crucially considered to select an optimal Wc.

The results are shown in Fig. (**5**). The curves of DSC exhibit an upward trend with the increase in the weights of the heart, whereas the MAE curves exhibit a downward trend. Both the separation of the heart and lung impedance images capability in terms of segmentation precision (DSC) and the performance of conductivity distribution learning (MAE) increase with the increase in the weight of the heart.

**Fig. (6):**
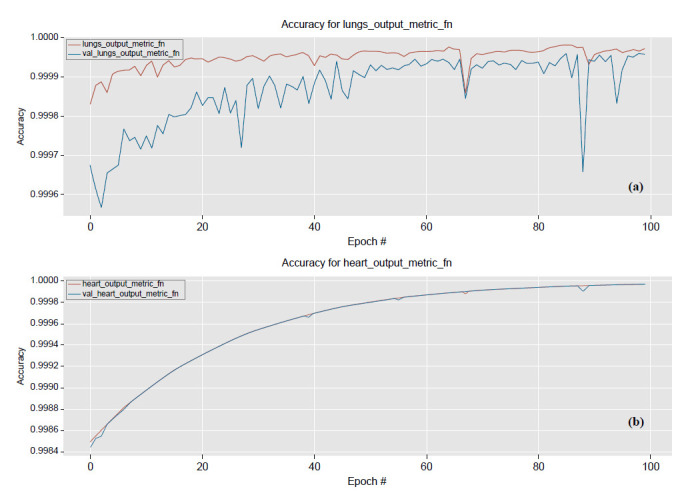
Performance progression according to the number of epochs with training and validation data under 2.0 and 1.0 of Wc, respectively. Illustration of (**a**) lung and (**b**) heart impedance image separation performance.

The model was trained under weights ratio Wc of 2.0 and 1.0, respectively, as shown in Fig. (**8a**). The curve of lung separation performance progression fluctuates between the training and validation data, and the convergent curves are not consistent (Fig. **6a**). In contrast, the heart separation performance progression on the training data matches the validation data, as shown in Fig. (**6b**). However, obtaining accurate heart and lung separation capability was the main objective of this study. This result can potentially be achieved by enhancing the heart data weighted loss contribution and proving the effectiveness of raising the weight of the heart.

Hence, the novel semi-Siamese U-Net model with a weight ratio Wc of 1.8 was the optimal parameter setting for the co-separation tasks. There is a trade-off between the performance and stability of the model, which makes the model more generalizable in real-world scenarios. Thus, the optimal version of the model was adopted for direct application in a real human EIT dataset to implement the separation of heart and lung functional impedance images. Excellent automatic separation results were obtained, as described in the next subsection.

### 3.2 Real human EIT Data Application

After the optimal hyperparameter selection, both segmentation performances greatly increased to values over 99%, produced consistent results in the validation dataset, and converged stably and rapidly. This means that the semi-Siamese U-Net structure with optimal parameterization weighting is capable of EIT imaging; however, the trained model must apply to real-world EIT imaging. Subsequently, the simulation-trained model with optimal Wc was directly applied to perform automatic EIT heart imaging reconstruction with publicly available real human data. The sequence images were reconstructed using the GREIT algorithm, as shown in Fig. (**7**) below. A series of 24 consecutive EIT images, approximately 3.3 s, from a healthy adult were used. The lung-induced impedance changes were depicted by the original EIT reconstruction algorithm, GREIT, while the heart-induced impedance changes were not presented in each frame, as shown in Fig. (**7**).

### 3.3 Heart Imaging Reconstruction

**Fig. (8):**
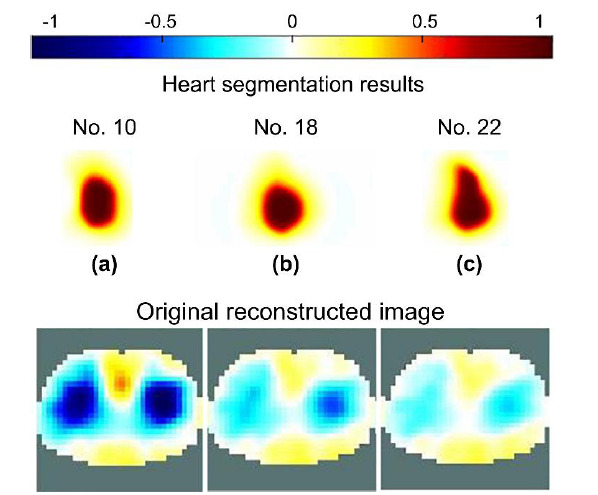
Heart segmentation results obtained using the optimal semi-Siamese U-Net model and comparison with the original EIT images. Frames (**a**) 10, (**b**) 18, and (**c**) 22 represent relatively high, middle, and low ventilation-induced impedance changes, respectively.

Three images, frames 10, 18, and 22, represent relatively high (Fig. **8a**), middle (Fig. **8b**), and low (Fig. **8c**) ventilation status, respectively. As the thorax EIT imaging limitation, the lung-induced impedance change dominates the EIT imaging. Therefore the different ventilation status was referenced to compare the heart imaging reconstruction results using optimal semi-Siamese U-Net reconstruction.

As shown in Fig. (**8**), the semi-Siamese U-Net approach effectively distinguishes the segmentation of the heart in consecutive images that show clear contours and morphology in each frame. This implies that the heart-induced impedance changes are not suppressed using the novel model.

In other words, the predicted heart-related impedance image is independent of that of the lung. The distinguished results of the separation of the heart-related impedance image obtained from the original EIT image can be attributed to the deep supervised learning of the optimal semi-Siamese U-Net. The results illustrate the feasibility of the semi-Siamese U-Net approach and its tailored architecture for heart segmentation in EIT.

## 4 DISCUSSION

The advantages of the lung EIT are that it is non-invasive and radiation-free, well-suited to be used at the bedside, and has functional imaging modality for continuous and long-term monitoring of the thorax, involving lung ventilation and perfusion simultaneously. However, separating the perfusion signal from the mixed EIT signal has its challenges, especially for patients with weak perfusion.

The optimal semi-Siamese U-Net model learned accurate separation and reconstruction of the functional images through only the simulation dataset. The model was not re-trained using real EIT data, yet it was able to reconstruct distinguishable functional impedance images in the real world. Experimental results revealed that the optimal semi-Siamese U-Net model is capable of simultaneous automatic lung and heart segmentation. Applying multi-weight loss and increasing heart weight achieved distinguished heart imaging results. This implies that the heart imaging reconstruction was trained under rigorous conditions, including penalization of the heart weight using the optimal hyperparameter, Wc, which is effective.

The novelty of this study is that no studies have reported separation from the perspective of simulations to reveal the effect of imaging at the center region. It also provides an easier method to generate efficient data for separating experiments. Moreover, U-Net architecture has not been applied to the separation of cardiac-related impedance changes in the EIT field until semi-Siamese U-Net was proposed in the experiments of this study.

The reconstruction results on real human EIT data in this study were evaluated qualitatively due to the absence of co-registered ground-truth segmentation or imaging modalities, such as CT or MRI. Consequently, quantitative metrics, such as DICE and MAE, could not be calculated for real human data, as there was no available anatomical reference to assess segmentation accuracy. However, qualitative observations of the reconstructed heart-related images showed consistent spatial and morphological features that align with expected cardiac anatomy across multiple frames. In future work, we aim to perform quantitative validation using datasets that include paired EIT and anatomical imaging (*e.g*., CT) [34] or manually annotated clinical labels to enable rigorous quantitative assessment of real patient data.

Recent advances in transformer-based segmentation architectures (*e.g*., D2PAM, Tri-M2MT, XAI-RACapsNet, and Dual-3DM3AD) have shown promising results in signal disentanglement and modality fusion in complex biomedical imaging [35-37]. In particular, D2PAM's dual attention strategy and Tri-M2MT's multi-transformer learning highlight the importance of context-aware features in multi-task learning. XAI-RACapsNet further enhances model explainability through relevance-aware capsule networks and ROI segmentation. Meanwhile, Dual-3DM3AD integrates mixed transformers with triplet pre-processing to improve semantic segmentation and early diagnosis in neurodegenerative diseases. While these models target distinct clinical domains, they collectively underscore the growing relevance of attention-guided and complexity-aware architectures. The semi-Siamese U-Net employed in this study contributes to this space by offering a lightweight yet effective design for signal separation in low-resolution EIT images, establishing a foundation for future integration of attention-based mechanisms in cardiopulmonary monitoring applications.

Future work will focus on domain adaptation *via* transfer learning or unsupervised adaptation to align the model with hospital EIT systems, including motion correction and real-time inference optimization.

## 5 STUDY LIMITATIONS

The proposed semi-Siamese U-Net model was trained exclusively on FEM-based EIT simulation data. Although promising results were observed when applying the model to real human data, the absence of fine-tuning using actual clinical datasets limits the model’s generalizability and robustness in practical scenarios. Biological variability, such as anatomical differences, disease conditions, and patient motion artifacts, were not accounted for in the simulation setup. Additionally, the model’s segmentation accuracy has not been validated using co-registered ground-truth imaging (*e.g*., CT or MRI) or physiological benchmarks.

Future work should focus on validating this model with animal experiments or clinical datasets that incorporate ground-truth references and pathological variability. Fine-tuning or domain adaptation with real-world EIT data, including CT-guided EIT validation, is essential before clinical integration. This step is critical for ensuring that the proposed method can support PEEP titration or perfusion monitoring in real-world Intensive Care Unit (ICU) environments.

## CONCLUSION

This study presents a novel semi-Siamese U-Net framework for reconstructing heart-related functional impedance images from lung EIT data, trained using FEM-based simulations. The approach enhances cardiac imaging resolution and achieves simultaneous segmentation of the lung and heart regions with high accuracy, as demonstrated by Dice scores and MAE metrics. To the best of our knowledge, this is the first study to employ a dual-decoder U-Net architecture for cardiac signal separation in EIT.

From a translational standpoint, the proposed method offers a foundation for enabling bedside monitoring of both ventilation and perfusion in patients with ARDS or other critical conditions. Integrating this model with clinical workflows could improve individualized PEEP titration and facilitate continuous assessment of cardiopulmonary function. However, further validation of clinical datasets and real-time implementation studies is necessary to support deployment in routine critical care settings.

## Figures and Tables

**Fig. (3):**
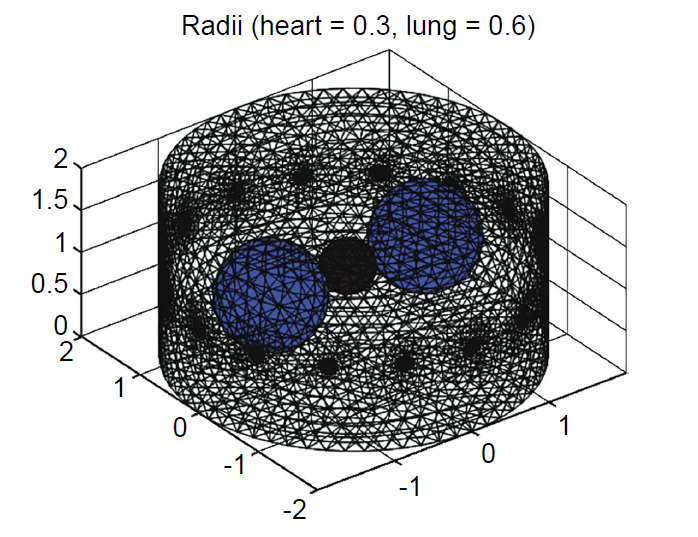
FEM-based phantom for EIT simulation built *via* EIDORS.

**Fig. (4a-f):**
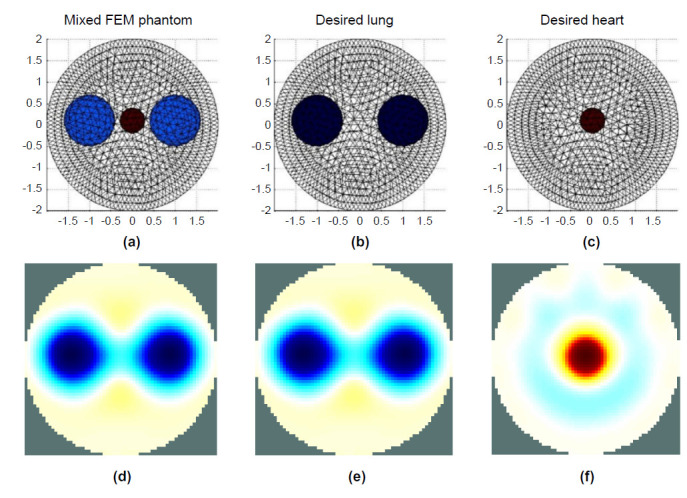
FEM-based phantoms and corresponding reconstructed EIT image for generating pair datasets.

**Fig. (5):**
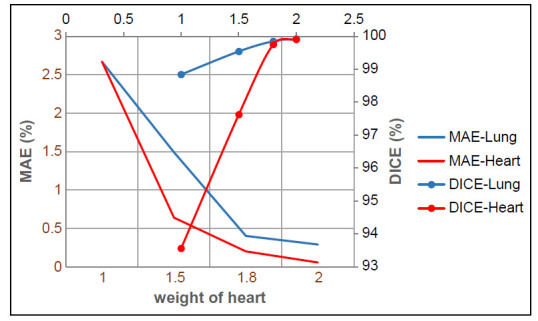
Illustration of semi-Siamese U-Net model performance with different weights of the heart, Wc. The evaluation of the semi-Siamese models on the test set based on metrics MAE and DSC.

**Fig. (7):**
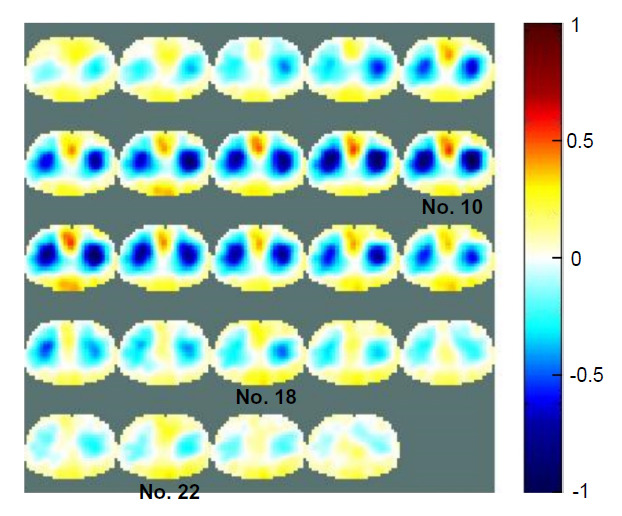
The real human EIT images reconstructed by GREIT (sampling rate: 7 Hz).

**Table 1:** Resistivity of different tissues.

**Tissue**	**Resistivity (ρ, Ωm)**
Blood	1.5
Heart	1.6 to 4.3
Lung (end of expiration)	7.2
Lung (end of inspiration)	23.6
Fat	27.2

**Table 2:** Summary of the semi-Siamese U-Net characteristics.

**Functions**	**Operation**	**Proposed Modifications**
Join learning under the same scales	Individual learning pathways	One-encoder and duo- decoders
Rigorous learning	Multi weighted loss	New loss function

**Table 3:** Hyperparameters used in this study.

**Optimizer**	**Adam**	
Convolutions	Semi-Siamese U-Net	
Parameterization new losses	Weight constants 1	W_heart_
	Weight constants 2	W_lung_

**Table 4:** Parameters of the thorax EIT simulation and FEM-based phantoms used.

**Electrodes**	**Characteristics**	**Spheres Models**	
16-electrode ring	Pair drive (adjacent): 1 mA Algorithm: GREIT	Both sides	Middle
		Conductivity: 0.5 S m^−1^	Conductivity: 2 S m^−1^
		Radii: 0.3–0.6 (unit: arbitrary)	Radii: 0.1–0.3 (unit: arbitrary)

**Table 5:** Results of using different ratios of weight constants in loss function in semi-Siamese U- Net and comparison with baseline model, Classical U-Net.

-	**Semi-Siamese U-Net model**							
W_c_ = 1.0	Relative Improvement	W_c_= 1.5	Relative Improvement	W_c_= 1.8	Relative Improvement	W_c_= 2.0	Relative Improvement
DICE (%)	93.56	5.18	97.62	9.24	99.75	11.37	99.90	11.53
MAE (%)	2.67	0.69	0.64	2.72	0.20	3.16	0.056	3.304
	*Baseline model: Classical U-Net							
DICE (%)	88.38	-	-	-	-	-	-	-
MAE (%)	3.36	-	-	-	-	-	-	-

**Note:** *Relative improvement: (Score_ model - Score_baseline model).

## Data Availability

All relevant data are available within the Dryad repository at DOI: 10.5061/dryad.47d7wm3c3. The public EIT data of a healthy adult provided by EIDORS was used to test the ability of the model to reconstruct EIT heart imaging. The public data are available from the EIDORS website: https://sourceforge.net/p/eidors3d/code/HEAD/tree/trunk/eidors/sample_data/montreal_data_1995.mat.

## LIST OF ABBREVIATIONS

EIT: Electrical Impedance Tomography
PEEP: Positive End-Expiratory Pressure
ARDS: Acute Respiratory Distress Syndrome
